# Supplementary figures and images for: Huntingtin preserves mitochondrial genome integrity in neurons, which is impaired in Huntington’s disease
Source: bioRxiv. 2025 Jul 24:2025.07.24.666629. Preprint. [Version 1] doi: 10.1101/2025.07.24.666629 (PMC12330591; doi:10.1101/2025.07.24.666629)

Figure-S1

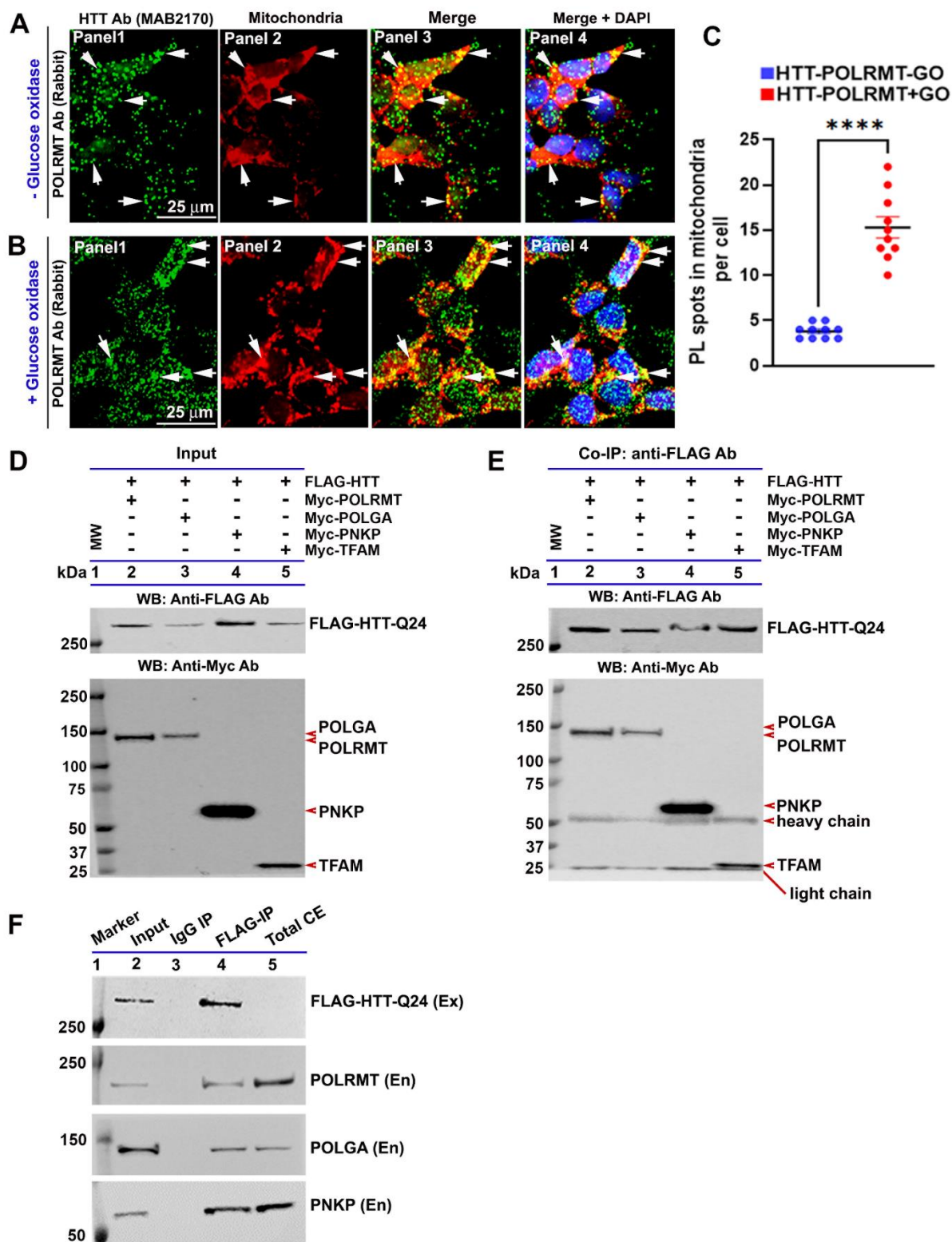

Figure-S2

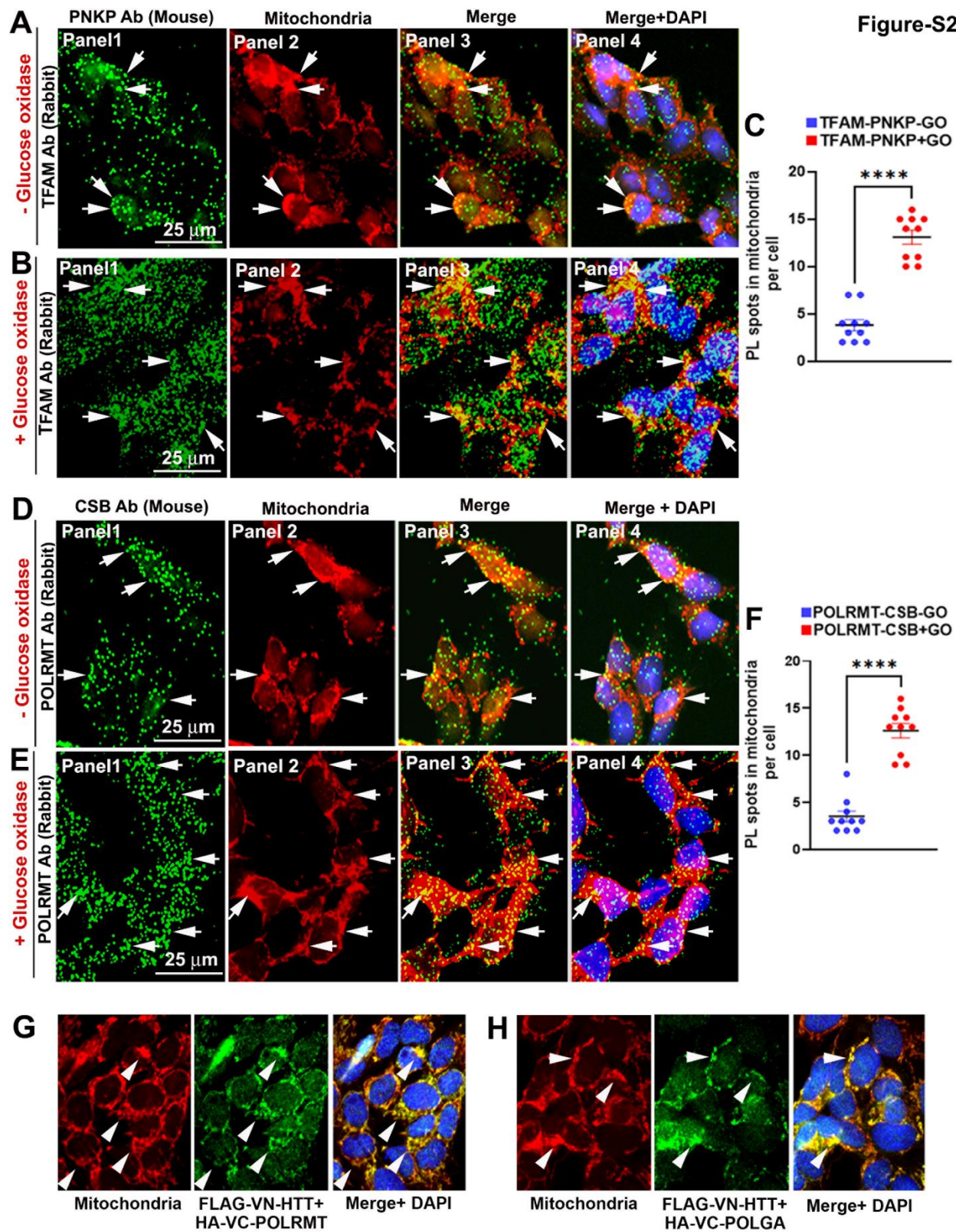

Figure-S3

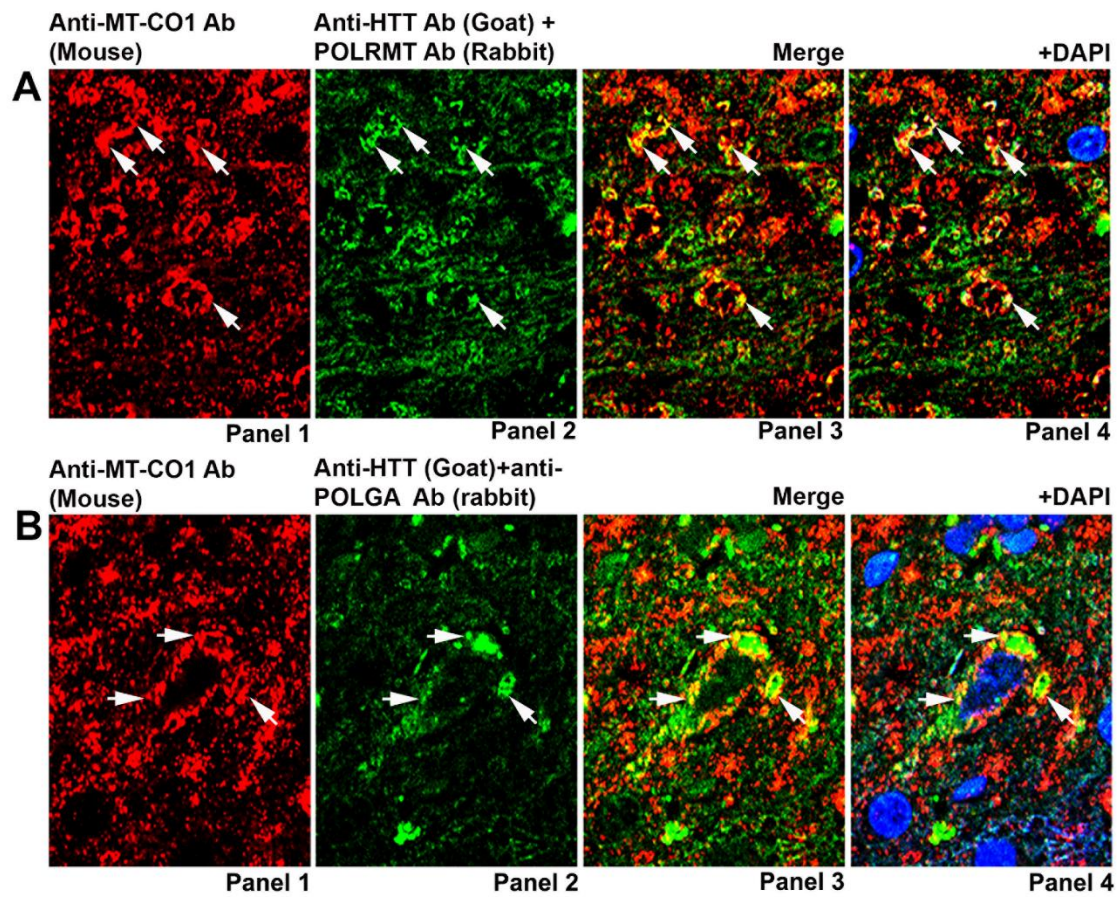

Figure S4

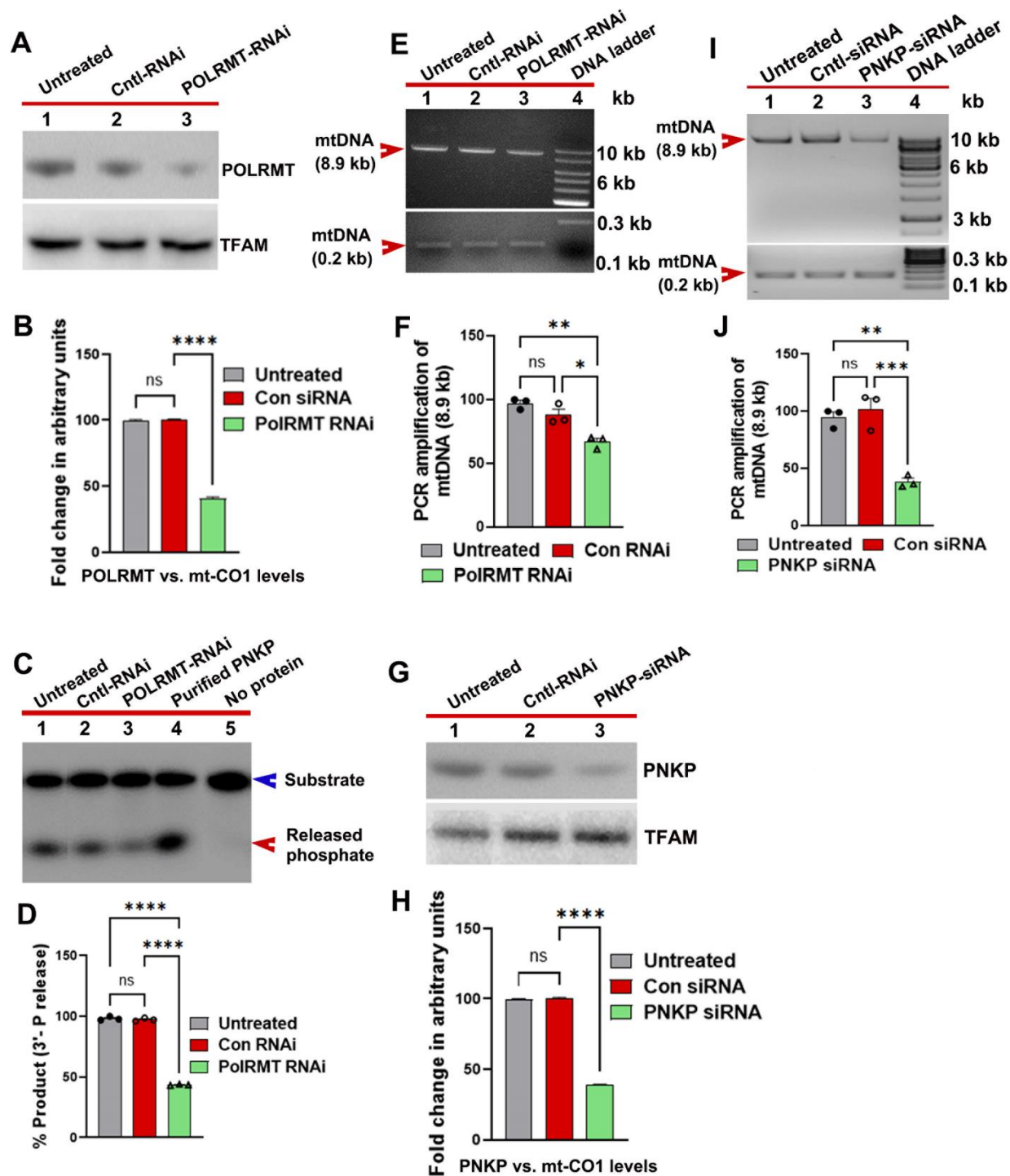

Figure-S5

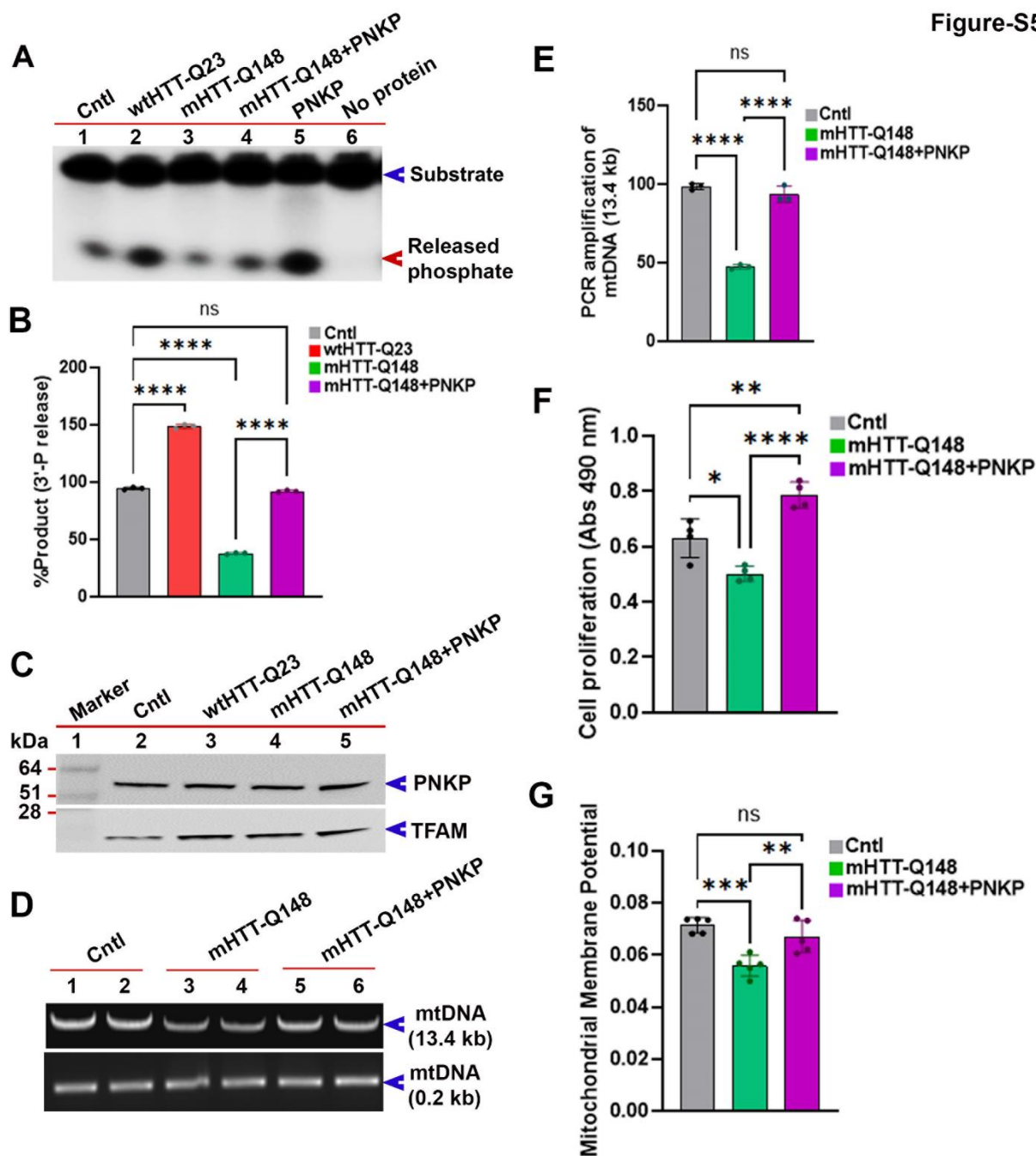

Figure-S6

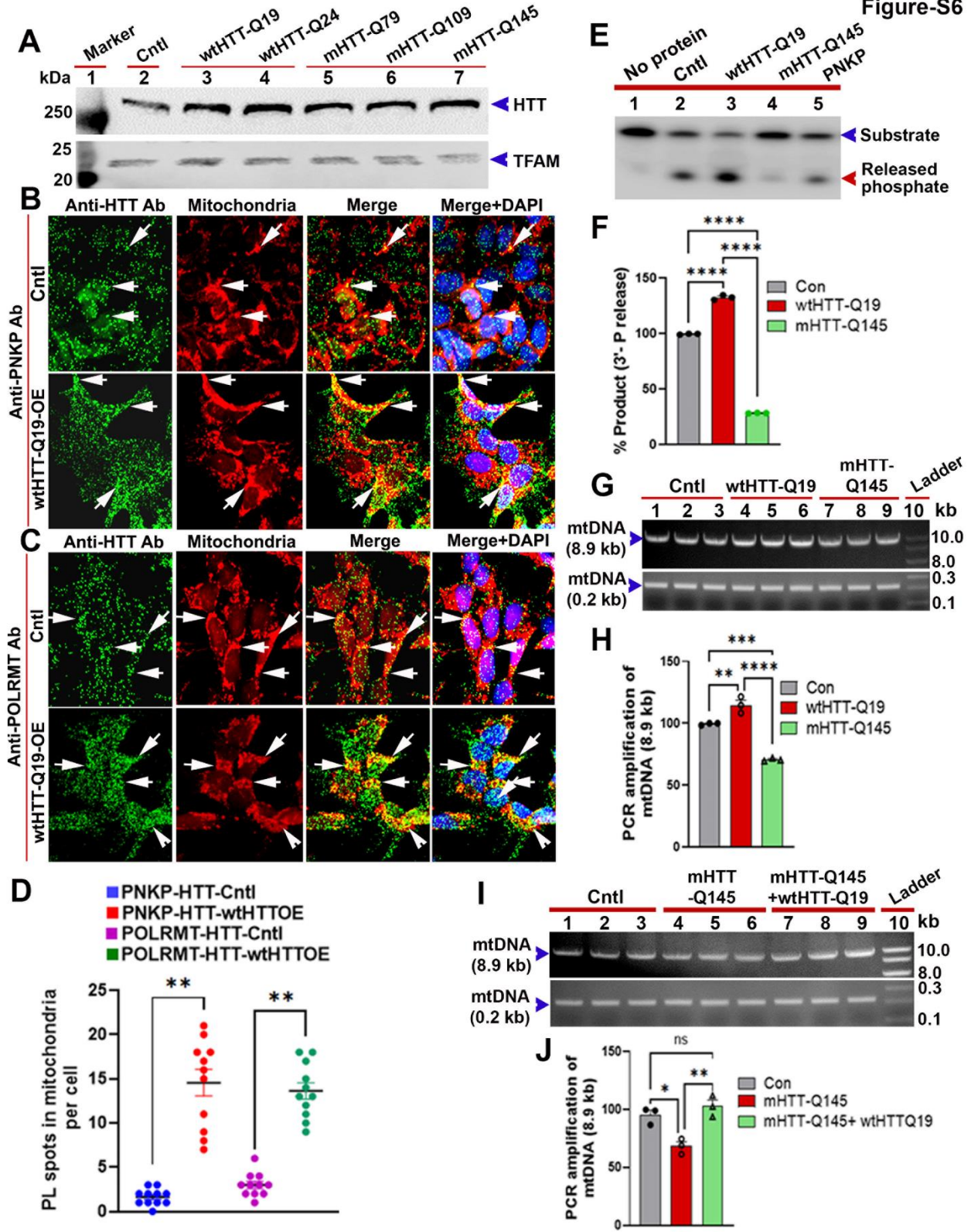

Figure-S7

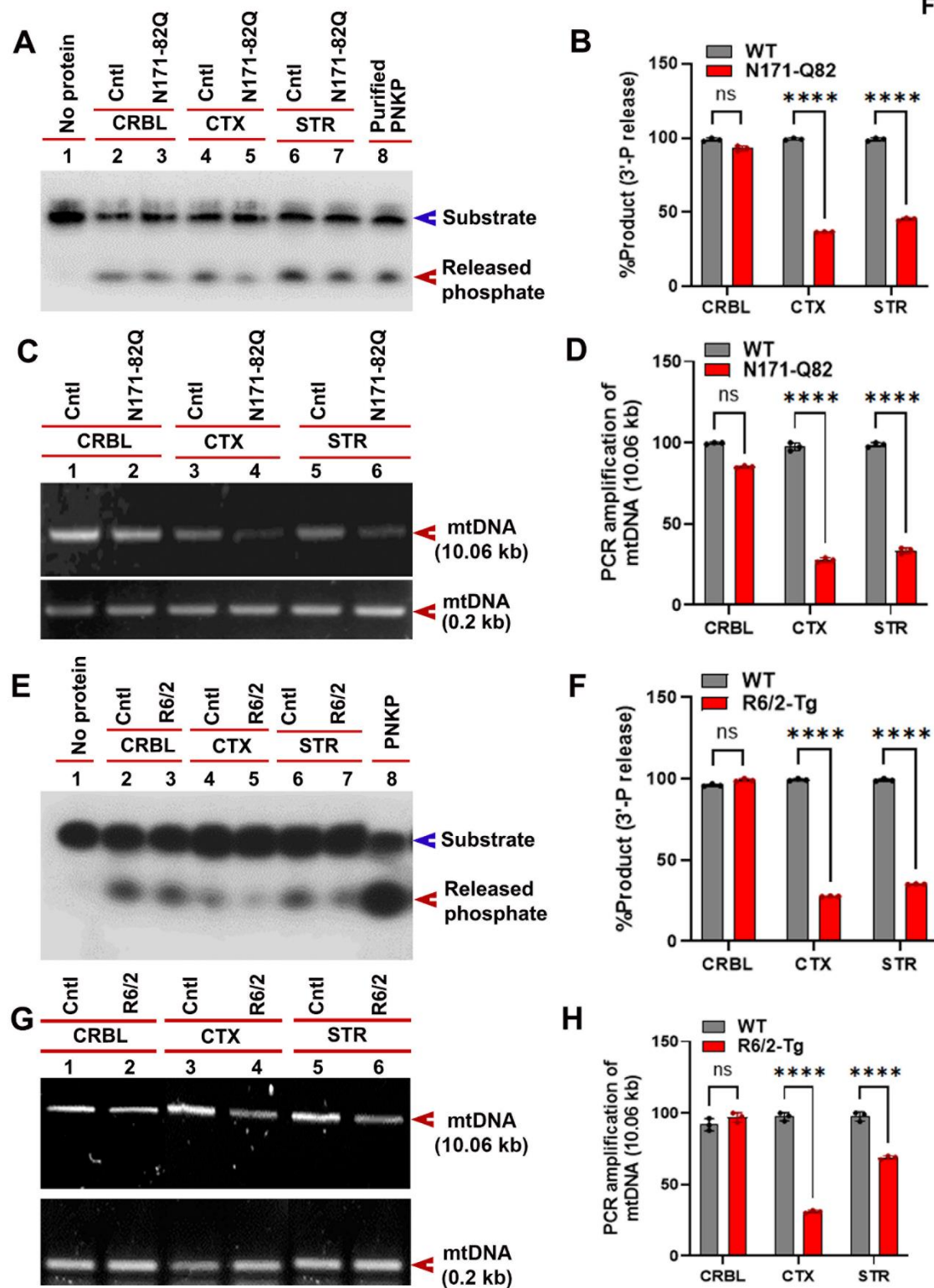

Supplement: Supplement 1 — Supplementary figure 1: HTT forms macromolecular complex with the mitochondrial transcription complex components in mitochondria. (A). PLA in control (-GO) SH-SY5Y cells with anti-HTT mouse monoclonal Ab (MAB2170; Millipore-Sigma) and anti-POLRMT rabbit polyclonal Ab (GTX105137; Genetex). (B). PLA in GO treated SH-SY5Y cells with an anti-HTT mouse monoclonal Ab (MAB2170; Millipore-Sigma) and with anti-POLRMT rabbit polyclonal Ab (GTX105137; Genetex). (C). Relative PLA signals showing substantial increased interaction of endogenous HTT with POLRMT within the mitochondria in GO-treated (+GO) cells compared to control (−GO). Data represent mean ± SD; ****p<0.0001. (D). Plasmids encoding FLAG-tagged wtHTT-Q24 and Myc-tagged PNKP, POLRMT, POLGA or TFAM separately co-transfected into human HEK293 cells, cell- extracts isolated 48 hours post-transfection, and analyzed by WBs to detect the expressions of exogenous FLAG-tagged HTT and Myc-tagged PNKP, -POLRMT, -POLGA, and - TFAM (arrows). (E). Cell extracts isolated from the co-transfected HEK293 cells expressing FLAG-tagged HTT-Q24 (FLAG-HTT-Q24), and Myc-tagged PNKP, POLRMT, POLGA or TFAM. The extracts were IP’d with anti-FLAG Ab (F3165; Sigma), and FLAG ICs analyzed by WBs to detect exogenous Myc-tagged-POLRMT, -POLGA, -PNKP or - TFAM. IgG heavy chain and light chain shown by arrows. Lane 1: Protein molecular weight marker. (F). MEs isolated from SH-SY5Y cells constitutively expressing exogenous (Ex) FLAG-tagged HTT-Q24, and IP’d with anti-FLAG Ab (F3165; Millipore-Sigma). The FLAG IC analyzed by western blotting to detect endogenous (En) POLRMT, POLGA, PNKP. Lane 1: protein molecular weight marker; lane 2: Input; lane 3 IgG IP; lane 4: FLAG IP and lane 5: total cell extract (Total CE). Supplementary figure 2: PNKP and CSB are present in mtDNA repair complex. Proximity ligation assay (PLA) performed to assess interaction/association of PNKP and mitochondrial transcription factor TFAM, or mitochondrial RNA polymerase (POLR [file media-1.pdf]
